# Supplementary material for: Camera traps unable to determine whether plasticine models of caterpillars reliably measure bird predation
Source: PLoS One. 2025 Mar 6;20(3):e0308431. doi: 10.1371/journal.pone.0308431 (PMC11884695; doi:10.1371/journal.pone.0308431)
Supplement: S2 Fig — The power analysis results according to the three different datasets are the following: predation clues 5% - 10% predation evidence: 99.80% (range: 99.28, 99.98); clues 8% - 9% evidence: 99.90% (range: 99.44, 100.00); clues 9% - 9% evidence: 0.00% (range: 0.00, 0.37). (PDF) [file pone.0308431.s002.pdf]

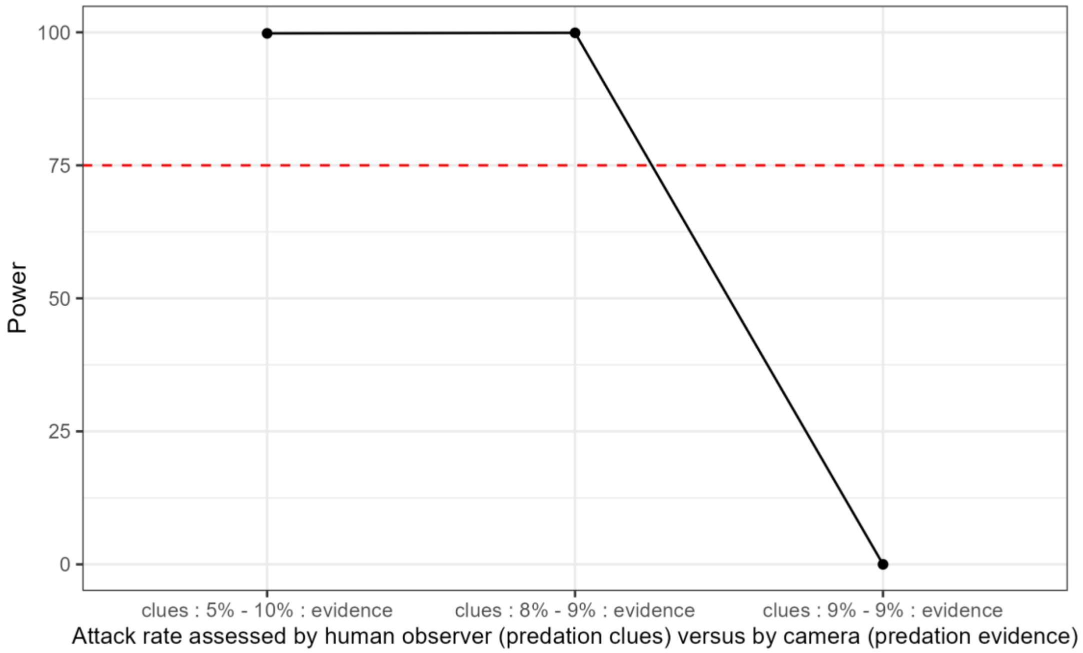

S2 Fig. The results of power analyses with 1000 simulations for the three different scenarios tested (hypotheses 2 and 4).

The power analysis results according to the three different datasets are the following: predation clues 5% - 10% predation evidence: 99.80% (range: 99.28, 99.98); clues 8% - 9% evidence: 99.90% (range: 99.44, 100.00); clues 9% - 9% evidence: 0.00% (range: 0.00, 0.37).
