# Supplementary material for: Camera traps unable to determine whether plasticine models of caterpillars reliably measure bird predation
Source: PLoS One. 2025 Mar 6;20(3):e0308431. doi: 10.1371/journal.pone.0308431 (PMC11884695; doi:10.1371/journal.pone.0308431)
Supplement: S3 Fig — The power analyses were based on the sample size of 420 caterpillars per fixed factor. The power analysis results according to the three different datasets are the following: model 5% - 10% corpse: 83.30% (range: 80.84, 85.56); model 5% - 20% corpse: 100.00% (range: 99.63, 100.00); model 10% - 20% corpse: 99.30% (range: 98.56, 99.72). (PDF) [file pone.0308431.s003.pdf]

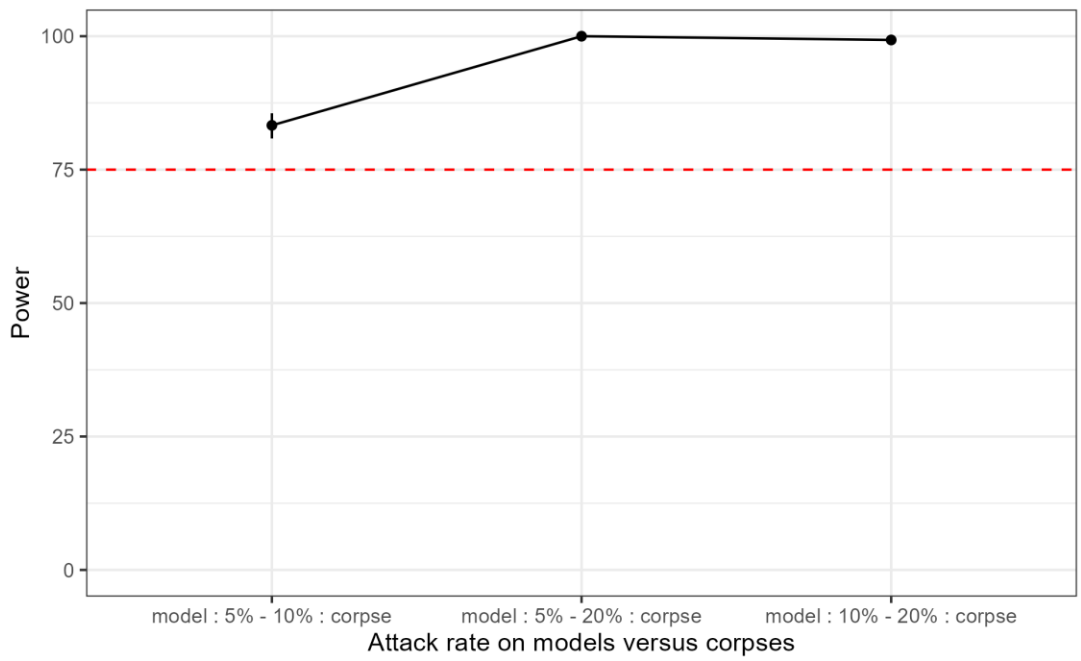

S3 Fig. The results of power analyses with 1000 simulations for the 3 different scenarios tested (hypothesis 3).

The power analyses were based on the sample size of 420 caterpillars per fixed factor. The power analysis results according to the three different datasets are the following: model 5% - 10% corpse: 83.30% (range: 80.84, 85.56); model 5% - 20% corpse: 100.00% (range: 99.63, 100.00); model 10% - 20% corpse: 99.30% (range: 98.56, 99.72).
