# Supplementary material for: Camera traps unable to determine whether plasticine models of caterpillars reliably measure bird predation
Source: PLoS One. 2025 Mar 6;20(3):e0308431. doi: 10.1371/journal.pone.0308431 (PMC11884695; doi:10.1371/journal.pone.0308431)
Supplement: S4 Fig — Camera traps photographs of potential predators of caterpillars. The blue squares correspond to MegaDetector detections with the associated confidence level. The animal species included are: the Long-tailed Tit (Aegithalos caudatus, S4a and S4ag Figs), the Tree Pipit (Anthus trivialis, S4c and S4k Figs), the Common Chiffchaff (Phylloscopus collybita, S4b and S4g Figs), the European Robin (Erithacus rubecula, S4d, S4f, S4j, S4o, S4p, S4q, S4s, S4ab, and S4ad Figs), the Eurasian Jay (Garrulus glandarius, S4e, S4l, S4r, S4y, S4af, S4ak, S4an, and S4ap Figs), the European Pied Flycatcher (Ficedula hypoleuca, S4h and S4i Fig.), the Red Squirrel (Sciurus vulgaris, S4m Fig), the Eurasian Blue Tit (Cyanistes caerulus, S4n and S4am Figs), the Great Tit (Parus major, S4t, S4v, S4w, S4aa, S4ac, S4ah and S4ai Figs), the Common Chaffinch (Fringilla coelebs, S4u, S4x, S4ae, S4aj, S4al, S4ao and S4aq Figs), unidentified rodents (S4z Fig), and the Song Trush (Turdus philomelos, S4ar Fig). (PDF) [file pone.0308431.s004.pdf]

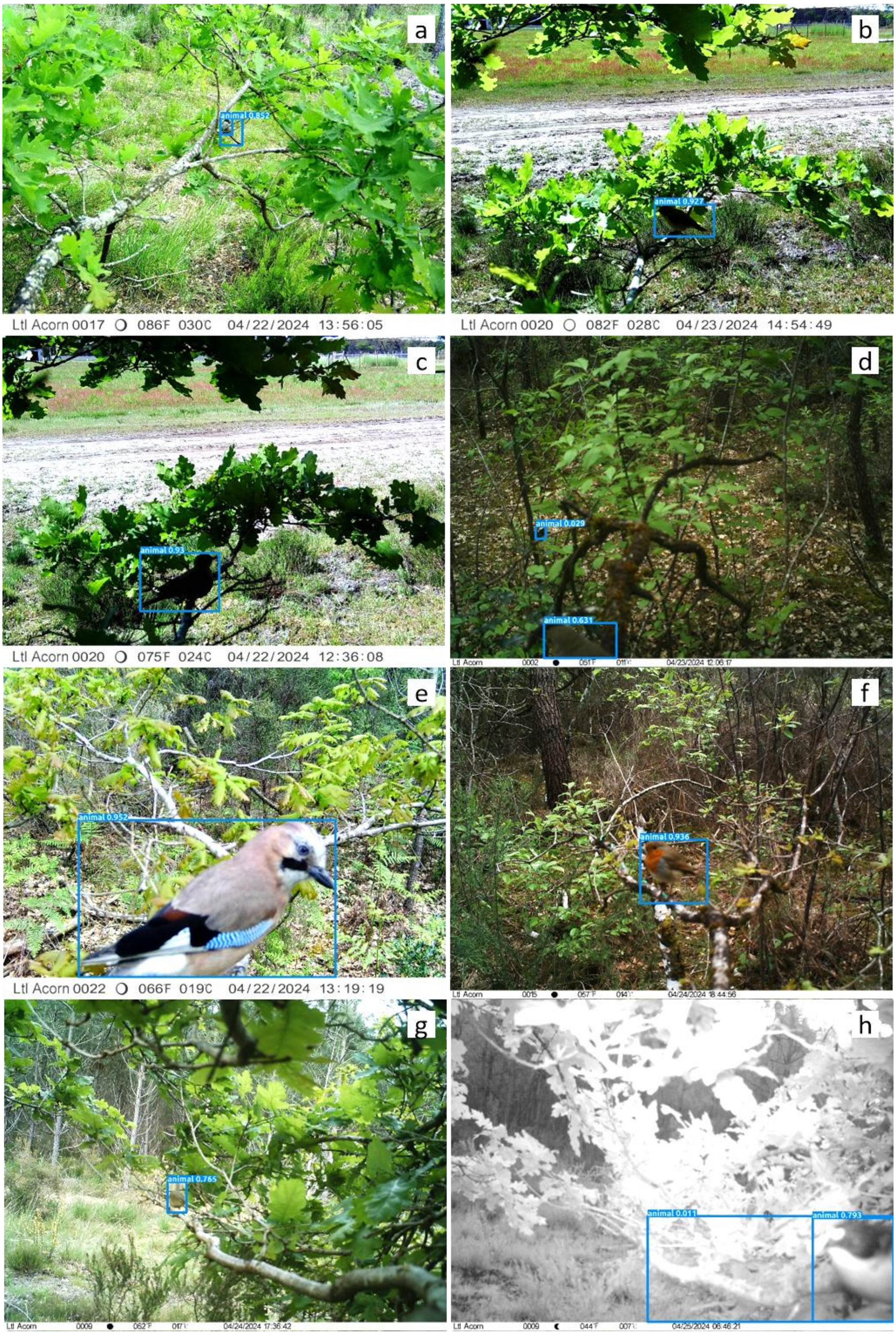
a
Ltl Acorn 0017 086F 030C 04/22/2024 13:56:05
b
Ltl Acorn 0020 082F 028C 04/23/2024 14:54:49
c
Ltl Acorn 0020 075F 024C 04/22/2024 12:36:08
d
e
Ltl Acorn 0022 066F 019C 04/22/2024 13:19:19
f
g
h

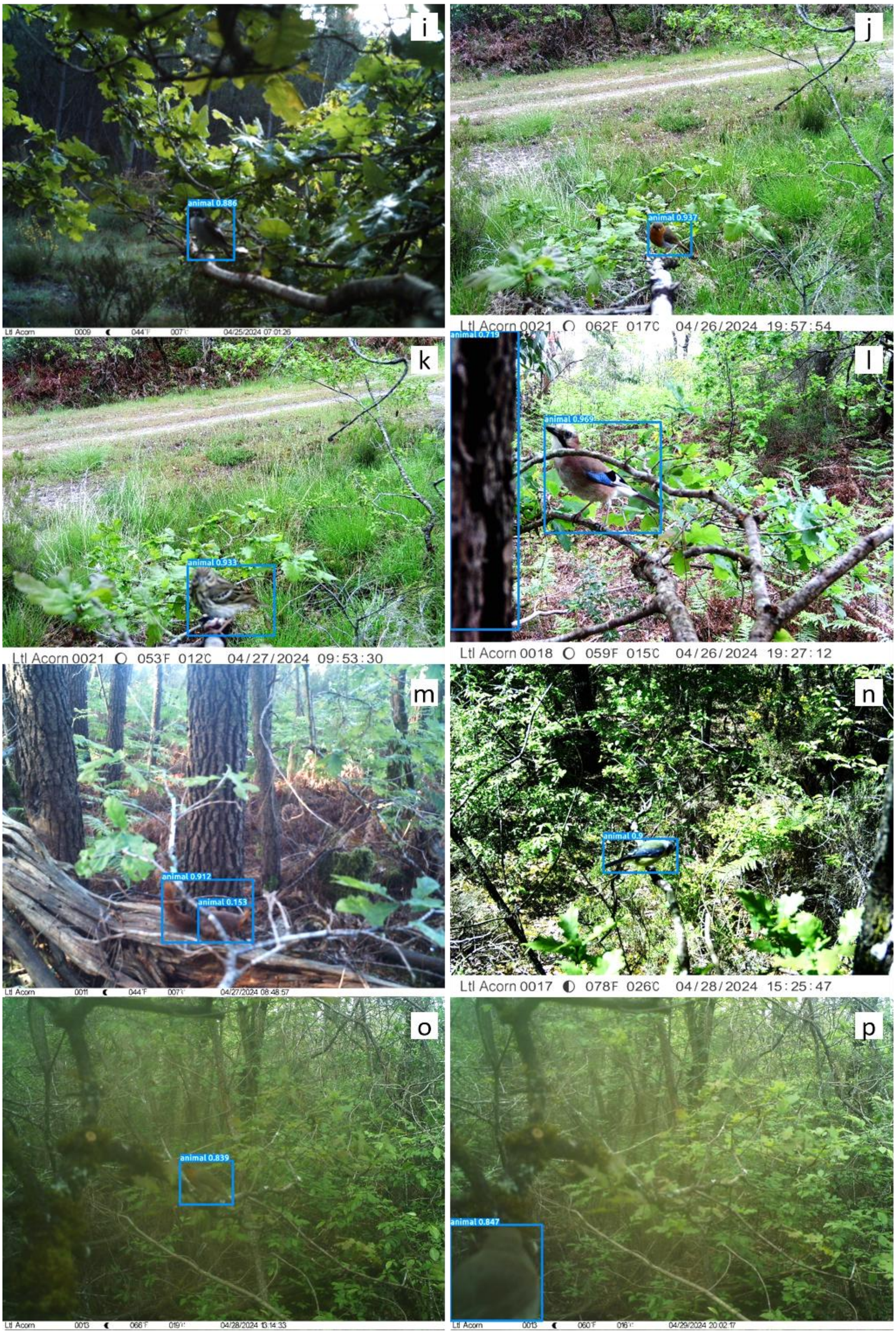
i
animal 0.886
j
animal 0.937
Ltl Acorn 0021 062F 017C 04/26/2024 19:57:54
k
animal 0.933
Ltl Acorn 0021 053F 012C 04/27/2024 09:53:30
l
animal 0.719
animal 0.969
Ltl Acorn 0018 059F 015C 04/26/2024 19:27:12
m
animal 0.912
animal 0.153
n
Ltl Acorn 0017 078F 026C 04/28/2024 15:25:47
o
animal 0.839
p
animal 0.847

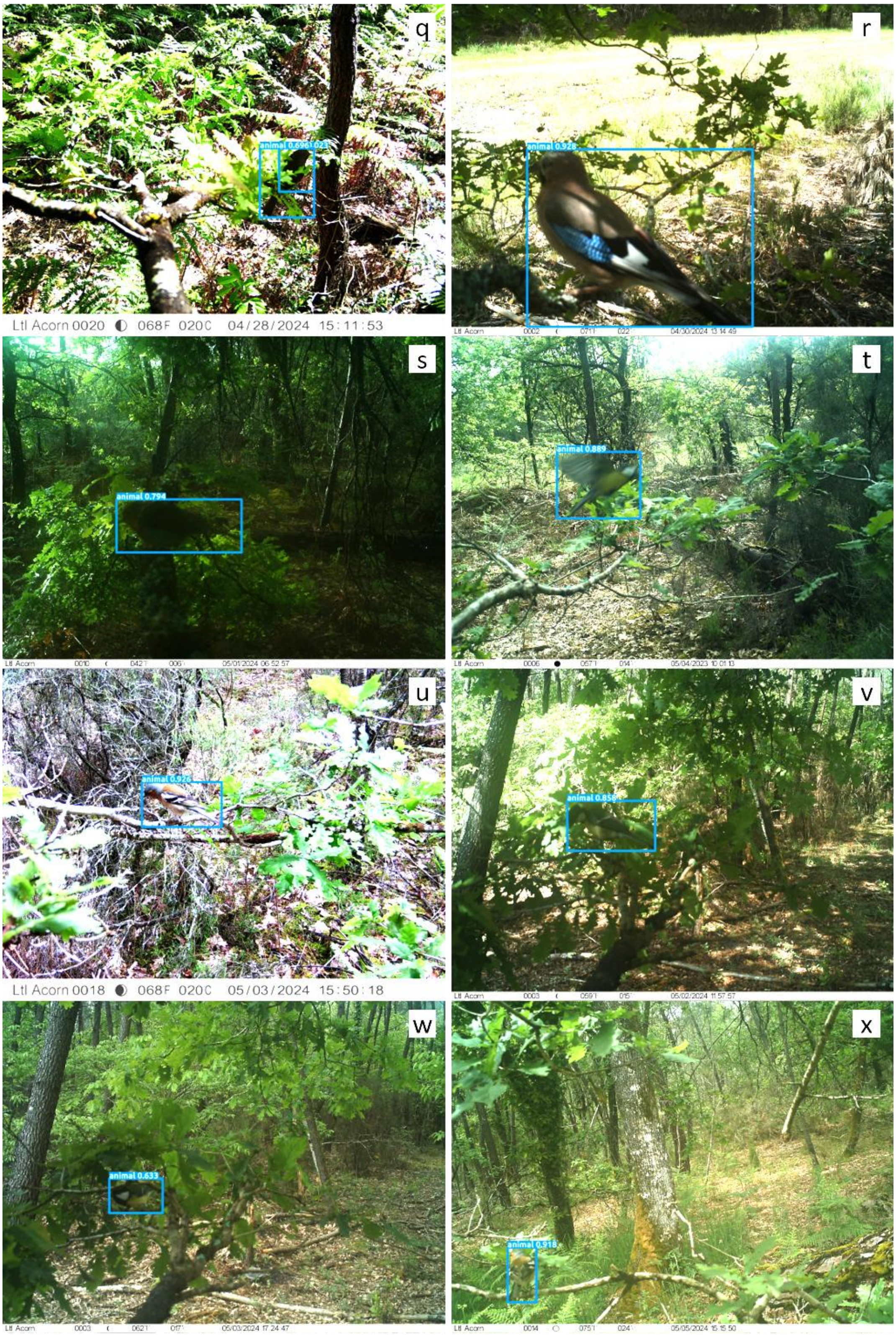
q
animal 0.696
Ltl Acorn 0020 068F 020C 04/28/2024 15:11:53
r
animal 0.928
s
animal 0.794
t
animal 0.889
u
animal 0.926
Ltl Acorn 0018 068F 020C 05/03/2024 15:50:18
v
w
animal 0.633
x
animal 0.918

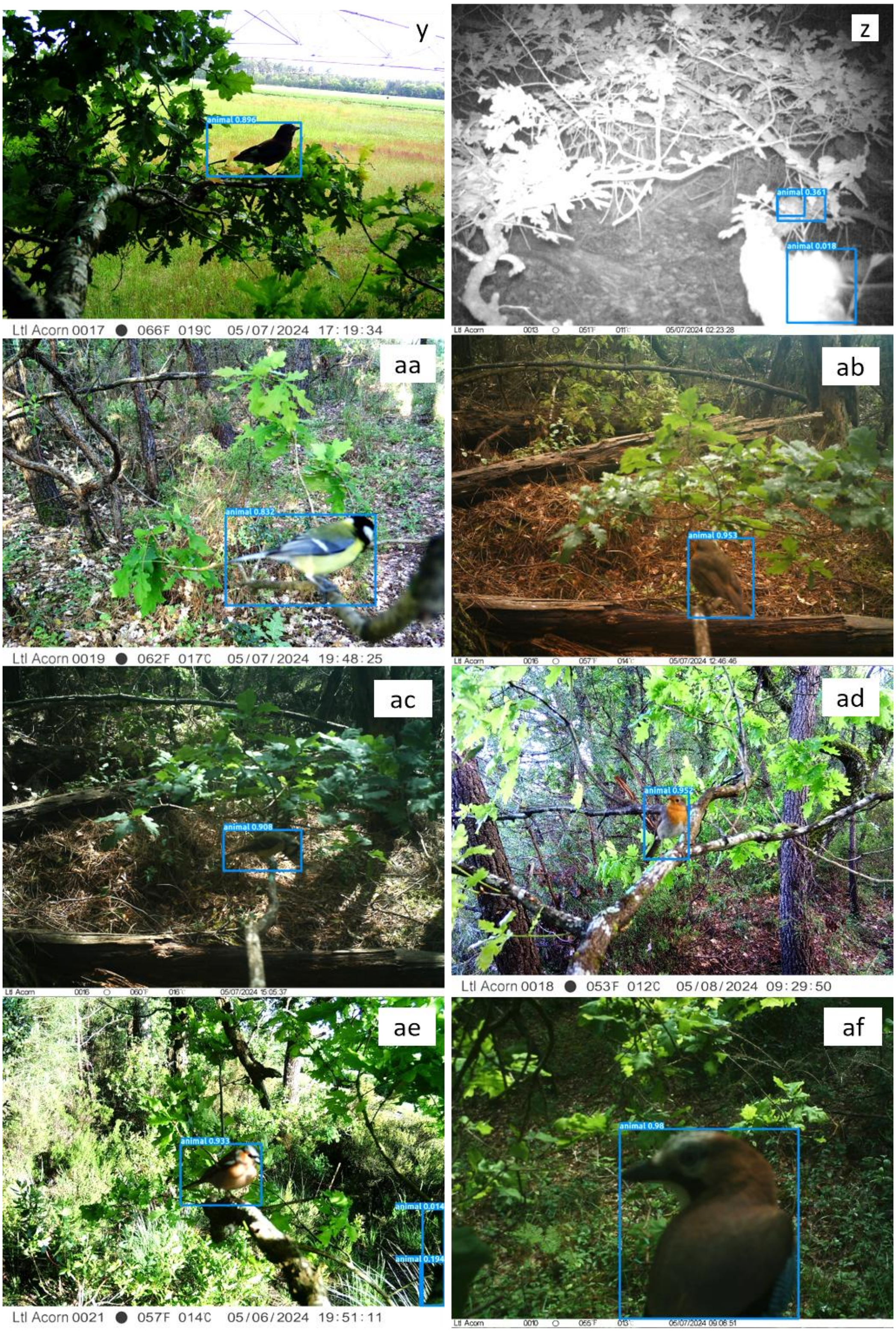
y
animal 0.896
Ltl Acorn 0017 066F 019C 05/07/2024 17:19:34
z
animal 0.361
animal 0.018
aa
animal 0.832
Ltl Acorn 0019 062F 017C 05/07/2024 19:48:25
ab
animal 0.953
ac
animal 0.908
ad
Ltl Acorn 0018 053F 012C 05/08/2024 09:29:50
ae
animal 0.933
Ltl Acorn 0021 057F 014C 05/06/2024 19:51:11
af
animal 0.98

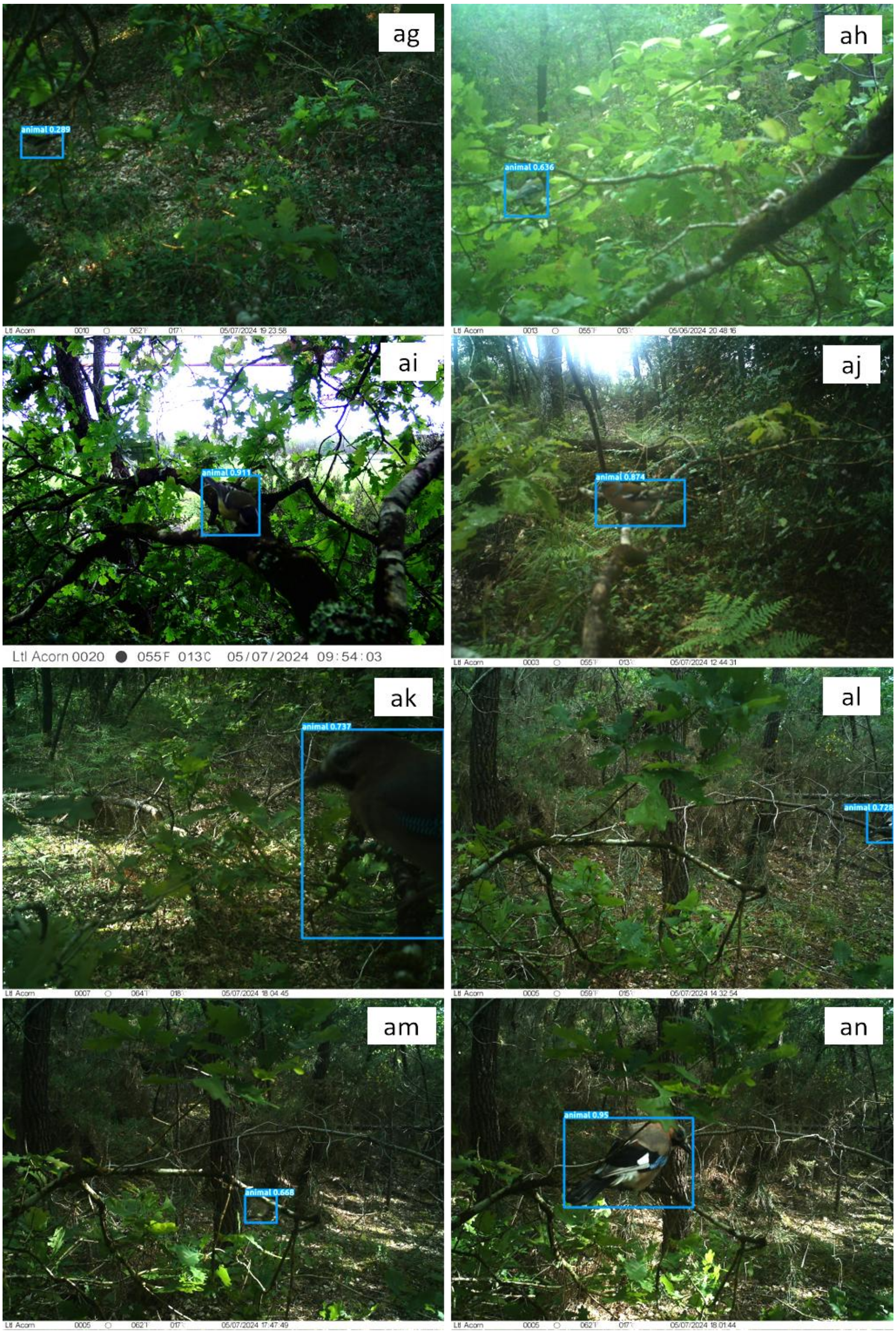
ag
animal 0.289
ah
animal 0.636
ai
animal 0.911
Ltl Acorn 0020 055F 013C 05/07/2024 09:54:03
aj
animal 0.874
ak
animal 0.737
al
animal 0.728
am
animal 0.668
an
animal 0.95

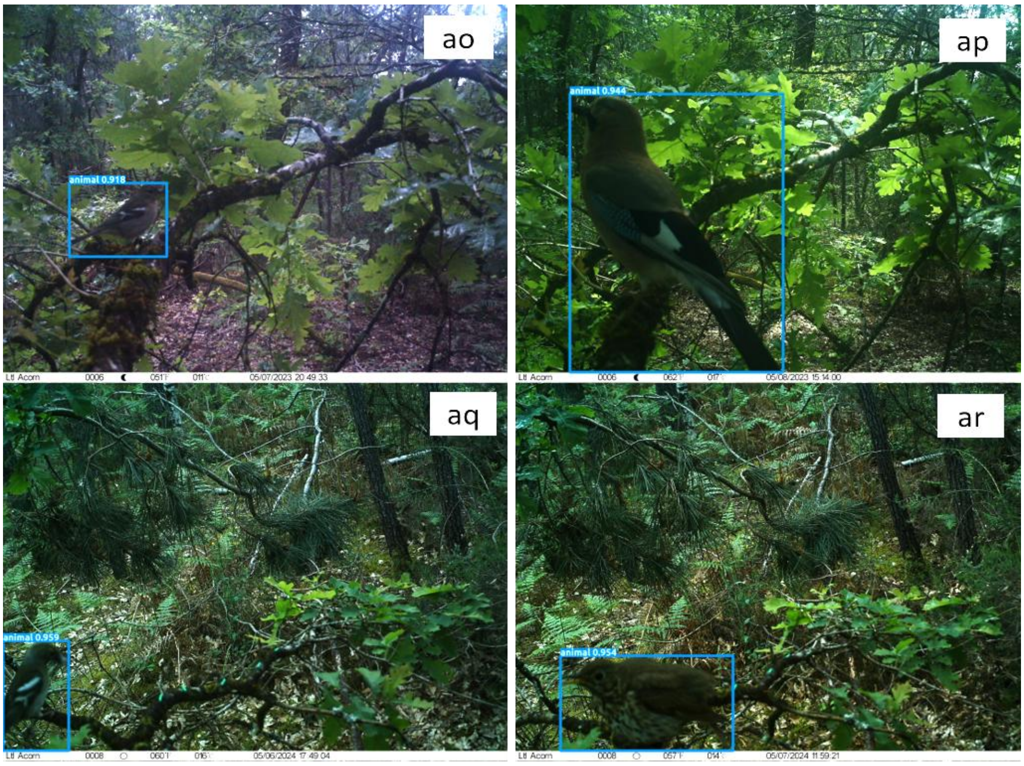

S4 Fig. All detected birds by MegaDetector AI software.
Camera traps photographs of potential predators of caterpillars. The blue squares correspond to MegaDetector detections with the associated confidence level. The animal species included are: the Long-tailed Tit (*Aegithalos caudatus*, S4a and S4ag Figs), the Tree Pipit (*Anthus trivialis*, S4c and S4k Figs), the Common Chiffchaff (*Phylloscopus collybita*, S4b and S4g Figs), the European Robin (*Erithacus rubecula*, S4d, S4f, S4j, S4o, S4p, S4q, S4s, S4ab, and S4ad Figs), the Eurasian Jay (*Garrulus glandarius*, S4e, S4l, S4r, S4y, S4af, S4ak, S4an, and S4ap Figs), the European Pied Flycatcher (*Ficedula hypoleuca*, S4h and S4i Fig.), the Red Squirrel (*Sciurus vulgaris*, S4m Fig), the Eurasian Blue Tit (*Cyanistes caerulus*, S4n and S4am Figs), the Great Tit (*Parus major*, S4t, S4v, S4w, S4aa, S4ac, S4ah and S4ai Figs), the Common Chaffinch (*Fringilla coelebs*, S4u, S4x, S4ae, S4aj, S4al, S4ao and S4aq Figs), unidentified rodents (S4z Fig), and the Song Trush (*Turdus philomelos*, S4ar Fig).
