# Supplementary material for: Camera traps unable to determine whether plasticine models of caterpillars reliably measure bird predation
Source: PLoS One. 2025 Mar 6;20(3):e0308431. doi: 10.1371/journal.pone.0308431 (PMC11884695; doi:10.1371/journal.pone.0308431)
Supplement: S5 Fig — From top to bottom, the families, genera, or species are: Cydalima perspectalis (non-native species), Geometridae family, Tenthredinidae family (likely of Caliroa genus), Tenthredinidae family (likely of Periclista genus), Operopthera brumata. Scale in cm. (PDF) [file pone.0308431.s005.pdf]

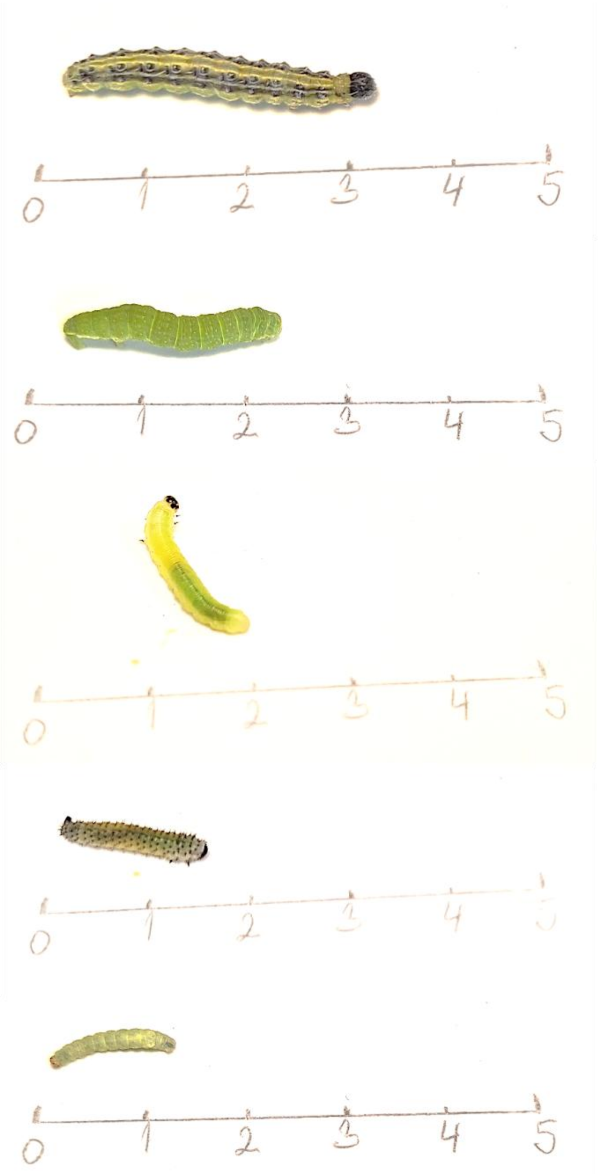

S5 Fig. Photographs of larvae found in the study area.

From top to bottom, the families, genera, or species are: *Cydalima perspectalis* (non-native species), Geometridae family, Tenthredinidae family (likely of Caliroa genus), Tenthredinidae family (likely of Periclista genus), *Operopthera brumata*. Scale in cm.
